# Acknowledgement to Referees

**DOI:** 10.1007/s13205-013-0193-6

**Published:** 2013-12-31

The Chief Editors and the Editorial Board thank the reviewers’ listed below for their contribution and time-consuming efforts in refereeing papers submitted to 3 Biotech in 2013. Their timely response and critical comments have not only assisted us in the selection of high-quality scientific work, but their critique is also appreciated by authors as it frequently assists them to improve the style and content of their publications.

A. Aboussekhra

H. Abulreesh

V. Acosta-Martinez

R. Agarwal

A. Aksyuk

A. Al-Halees

N. Al-Khalifah

I. Al-Saleh

E. Albertó

G. Alsbeih

N. Alsouhibani

A. AlZahrani

N. Annamalai

J. Aoki

A. Arya

S. Bagatharia

B. Bajaj

V. Bajpai

A. Best

T. Bhalla

P. Bhargava

A. Bhargava

M. Borowitzka

J. Bowen

M. Brochetto-Braga

C. Brown

V. Caffier

M. Camassola

D.J. Carrier

S. Chadha

Y. Chao

D. Chatanta

F. Chen

H. Chen

J. Chen

S. Chen

E.J. Cheong

A. Choubay

C. Chu

R. Colasanti

G. Coman

D. Cowan

B. Crother

Z. Dai

S. Das

A. Das

A. Debez

K. Dhar

S. Dhiman

C. Dolinski

N. Dzimiri

J. Edirisinghe

A. El Hadrami

A. El-Bondkly

K. Elmeer

G. Espín

J. Estelrich

F. Federici

J. Feng

L. Fki

R.S. Fougat

R. Gaur

T. Geng

S. Gerdes

P. Gladieux

Y. Gogorcena

A. Goyal

T. Greene

M. Greenway

E. Grohmann

V. Gupta

V.K. Gupta

G. Gutierrez-Sanchez

P. Hall

P. Hallenbeck

P. Hanmoungjai

K. Hata

K. Hawboldt

A. Hawwari

Z. He

Y. He

E. Hitti

R. Holmes

M. Hu

H. Huang

J. Huang

Z. Huang

N. Huda

M. Hurst

F. Imtiaz Ahmad

T. Imura

Y. Ishii

M. Jadhav

N. Jain

P. Jain

D. Jha

Y. Ji

W. Jiang

M. Johns

S. Johri

J. Jose

M. Joshi

S. Joshi

H. K

A. Karagouni

R. Kasana

P. Kasera

N. Kaushik

D. Kennedy

Md. S. Khan

R. Kharwar

S.-K. Kim

K. Kirimura

Y. Kolekar

E. Kothe

P.N. Krishnan

S. Kumar

P. Kumar

R. Kumar

A.A. Kwaasi

V. Ladero

V. Lakshmanan

R. Lal

S.K. Lattoo

J. Leach

D. Li

L. Li

K.-S. Ling

C. Liu

J. Liu

X. Lu

E. Luepromchai

J. Maity

N. Mandal

A. Manimaran

S. Mansoor

G. Markx

I. Marova

A. Maruyama

T. Mechichi

S. Mehetre

A. Mittal

G. Monard

S. Moukha

D. Mousdale

P. Mukherjee

A. Mukherjee

M. Nader

M.L. Narasu

D. Navarro

N. Nawani

P. Negi

Dr. T.B. Ng

T. Noguer

R. Norton

J. Novak

B. Padder

P. Panesar

S. Papanikolaou

B. Patel

B. Patra

C. Peña

A. Philippoussis

G. Phillips

C. Pineda-Vargas

C. Poizat

J. Prakashmaran

K. Prasad

R.E.L. Procópio

T. Pullaiah

L. Qing

E.K. Radhakrishnan

S. Raj

K.B. Ramachandran

N. Raman

J. Rao

R. Rawal

S. Rayner

G.R. Rout

G. Sablok

A. Satlewal

T. Sawidis

M. Schartl

S. Seaver

Z. Senwo

B. Sharma

A. Sharma

A. Sharma

D. Sharma

N. Sharma

Y. Shi

I.-L. Shih

M. Shoukri

O. Sidhu

S. Singh

H. Singh

H.B. Singh

S. Singh

U. Singh

H. Song

C. Song

A. Sonnevend

M. Srivastava

E. Taha

T.-C. Tan

Y. Tao

R. Thangavelu

G. Theodoropoulos

C.-G. Tian

R. Tiwari

A. Tripathi

R. Tu

S. Uddin

V. Vilara

R. Vinayak

A. Viswanathan

S. Waghmare

S. Walid

W. Wang

X. Wang

Y. Wang

Z. Wang

D. Watters

Q. Wu

M. Xian

S. Xiang

C. Xianzhong

J. Xing

Z. Xu

A. Xueqin

K. Yamanaka

Z. Yang

J. Yarbrough

J. Yi

I. Yoshikazu

S. Yu

G.H. Yue

X. Zhao

X.-H. Zhao

C. Zhen-ming

Y.-G. Zheng

Q. Zhou

D. Zuniga

